# Homocysteine Levels in Parkinson's Disease: Is Entacapone Effective?

**DOI:** 10.1155/2016/7563705

**Published:** 2016-07-17

**Authors:** Bilge Kocer, Hayat Guven, Selim Selcuk Comoglu

**Affiliations:** Department of Neurology, Diskapi Yildirim Beyazit Training and Research Hospital, 06110 Ankara, Turkey

## Abstract

Plasma homocysteine (Hcy) levels may increase in levodopa-treated patients with Parkinson's disease (PD) as a consequence of levodopa methylation via catechol-O-methyltransferase (COMT). Results from previous studies that assessed the effect of COMT inhibitors on levodopa-induced hyperhomocysteinemia are conflicting. We aimed to evaluate the effects of levodopa and entacapone on plasma Hcy levels. A hundred PD patients were enrolled to the study and divided into three treatment groups (group I: levodopa and/or dopamine agonists; group II: levodopa, entacapone, and/or a dopamine agonist; and group III: dopamine agonist alone). We measured the serum B12, folic acid, and Hcy levels in all patients. There were no statistically significant differences between groups in terms of modified Hoehn and Yahr stages, Unified Parkinson's Disease Rating Scale II/III, Standardized Mini-Mental Test scores, and serum vitamin B12 and folic acid levels. Plasma median Hcy levels were found above the normal laboratory values in groups I and II, but they were normal in group III. However, there was no statistically significant difference in plasma Hcy levels between groups. Our results showed that levodopa treatment may cause a slight increase in the Hcy levels in PD compared with dopamine agonists and that COMT inhibitors may not have a significant effect on preventing hyperhomocysteinemia.

## 1. Introduction

High levels of homocysteine (Hcy) are a known risk factor for vascular diseases and dementia in the general population [1, 2]. Plasma Hcy levels may increase as a result of genetic and acquired causes [3]. In terms of the genetic causes, a gene mutation exists that encodes the methylenetetrahydrofolate reductase (MTHFR) enzyme and is commonly encountered in the general population [3]. Plasma Hcy levels can also be affected by severe metabolic disorders, vitamin B12 and folic acid deficiency, and the use of vitamins and certain medications [3].

An increase in plasma Hcy levels has been reported in Parkinson's disease (PD) patients who were using levodopa. Total Hcy concentrations in the cerebrospinal fluid were also higher following levodopa therapy than before treatment and than in controls [4]. The catalysis of levodopa with the catechol-O-methyltransferase (COMT) enzyme results in the formation of S-adenosylhomocysteine (SAH), which hydrolyses to form Hcy [3].

Previous studies have indicated a relationship between Hcy and DNA damage, apoptosis, excitotoxicity, and oxidative stress, which are of great importance in neurodegeneration [3, 5]. Experimental studies have demonstrated that Hcy can be neurotoxic and excitotoxic to the substantia nigra. Furthermore, Hcy may be associated with dyskinesia, which is an indicator of possible neurodegeneration due to the disruption of the balance of striatal activity [6, 7]. Following* in vitro* and* in vivo* observations on the toxic effects of Hcy on dopaminergic neurons in the substantia nigra, some authors have suggested that hyperhomocysteinemia associated with levodopa can play a role in the progression of PD and the development of motor complications. Dyskinesia and motor fluctuations may be due to the toxic effects of Hcy [7, 8].

COMT inhibitors have been widely administrated to control motor complications such as “wearing off” in treatment of PD. Evidence of COMT inhibitors decreasing plasma Hcy levels that have been increased by levodopa in rats has had a pioneering influence on studies performed with COMT inhibitors on humans [9]. However, the results of studies performed to date have varied. While some studies have shown that COMT inhibitors can reduce plasma Hcy levels [10–12], this effect has not been demonstrated in others [13, 14]. Zesiewicz et al. examined 5 studies arising from Europe and USA and reported that the ability of COMT inhibition to reduce or prevent hyperhomocysteinemia induced by levodopa in PD patients may be attributed to differences in the vitamin status of the study participants. In addition, authors also indicated that, in patients with low or low-normal folic acid levels, levodopa administration is associated with a greater increase in Hcy and concomitant entacapone administration is associated with greater reduction in Hcy [15].

In this study, we evaluated the effects of various treatment options on plasma Hcy levels in idiopathic PD and investigated whether the addition of entacapone (a COMT inhibitor) to the treatment contributed to a reduction in plasma Hcy levels.

## 2. Methods

### 2.1. Subjects

For this prospective study, we enrolled one hundred patients (54 men, 54%) diagnosed with idiopathic PD according to the UK Parkinson's Disease Society Brain Bank Criteria [16]. The average age of the patients was 70 years (range 40–89). Patients with a severe metabolic disorder, a history of vitamin use, or secondary Parkinsonism were excluded from the study.

### 2.2. Study Design and Protocol

We recorded the following demographic data for all patients: age at disease onset, duration of disease, treatment regimes, levodopa dosage and duration of use, and entacapone dosage and duration of use. Then, we divided patients into three groups according to the type of treatment received.

Group I consisted of patients treated with levodopa and/or a dopamine agonist: 15 patients with levodopa alone and 43 patients with levodopa and dopamine agonists (17 pramipexole, 15 ropinirole, and 11 piribedil). The median duration of dopamine agonist treatment was 4 years (range 1–20).

Group II consisted of patients treated with levodopa, entacapone, and/or a dopamine agonist: 5 patients with levodopa and entacapone and 25 patients with levodopa, entacapone, and dopamine agonists (10 pramipexole, 10 ropinirole, and 5 piribedil). The median duration of dopamine agonist treatment was 7 years (range 1–24). Group II patients were treated with levodopa and entacapone simultaneously (levodopa-entacapone combined preparation).

Group III consisted of patients treated with a dopamine agonist alone (6 pramipexole, 4 ropinirole, and 2 piribedil). The median duration of dopamine agonist treatment was 2.5 years (range 1–12).

In previous studies, the effects of dopamine agonists on Hcy levels could not be demonstrated despite the fact that levodopa has been shown to increase plasma Hcy levels [10]. In this regard, we compare the plasma Hcy values of patients within groups I and II to patients within group III.

We used the “modified Hoehn and Yahr” (mHY) scale to evaluate the PD stage of each patient and the “Unified Parkinson's Disease Rating Scale” (UPDRS) Parts II and III, performed at the “on” period, to evaluate the severity of disease symptoms [17, 18]. Global cognitive status was evaluated using the “Standardized Mini-Mental Test” (SMMT).

For all patients, blood samples were taken from the peripheral vein and they were pooled into EDTA vacuum tubes in the morning after 12 hours of fasting and at 12-hour drug-free periods. Plasma Hcy levels were measured using the “high performance liquid chromatography with fluorescence detection” method according to Vester and Rasmussen [19]. The normal range for Hcy is considered between 5 and 15 *µ*mol/L [20]; however, evidence from large epidemiological studies indicates that Hcy levels lower than 15 *µ*mol/L are also associated with higher risk of vascular events [21–23]. Therefore, plasma levels for hyperhomocysteinemia were set higher than 12 *µ*mol/L, although plasma levels of Hcy above the 20 *µ*mol/L are also associated with greater risk.

Serum B12 and folic acid levels were analysed by automated electrochemiluminescent immunoassay (Elecsys 2010, Roche Diagnostics, Basel, Switzerland) [24]. Normal values range from 156 to 698 pg/mL for serum B12 and 3.1 to 17.5 ng/mL for serum folic acid.

All participants in this study provided informed consent. The study was carried out according to the Helsinki Declaration and was approved by the institutional ethics committee.

### 2.3. Statistical Analysis

Statistical analyses were performed using the SPSS Statistics Program (version 19, IBM, Chicago, IL). Initially, the normality of variables was studied. Analysis using the Kolmogorov-Smirnov test showed that the distribution of variables, except for folic acid, was not consistent with a normal distribution. We used nonparametric tests for the analysis of variables that were inconsistent with a normal distribution and parametric tests for the analysis of variables that were consistent with a normal distribution. The Chi-square test was used to study gender-related differences. The Mann-Whitney *U* test was used to analyse differences between subgroups of male/female populations with respect to plasma Hcy levels.

## 3. Results

Demographic data, clinical characteristics, and serum vitamin B12, folic acid, and Hcy levels are shown in Table 1.

We found statistically significant differences between treatment groups regarding age, disease duration, and age at disease onset (*p* = 0.04,* p* = 0.01, and* p* = 0.002, resp.). Specifically, group II patients (levodopa, entacapone, and/or dopamine agonist) were younger than group I patients (levodopa and/or dopamine agonist). Furthermore, while the disease duration was significantly longer in group II than in group III patients (dopamine agonist only), the age at disease onset was significantly lower in group II than that in both group I and group III patients. There were no differences between groups in terms of mHY stages, UPDRS Part II/III scores, and SMMT scores (Table 2).

Additionally, there were no statistically significant differences in serum B12 and folic acid levels between groups. While plasma median Hcy levels were above normal laboratory values (14 *µ*mol/L) in group I and II patients, they were normal in group III patients. However, there was no statistically significant difference in plasma Hcy levels between groups. Similarly, there was no statistically significant difference between group I and II patients in terms of the treatment dosages and duration of levodopa (Table 2).

We found plasma Hcy levels were statistically higher in men for all patients (*p* = 0.001). Intragroup plasma Hcy levels of male/female patients were also compared and there was no statistically significant difference between group I and group II; group I and group III; and group II and group III regarding plasma Hcy levels among men (*p* = 0.71, *p* = 0.16, and *p* = 0.62, resp.). Similarly there was no statistically significant difference between group I and group II; group I and group III; and group II and group III regarding plasma Hcy levels among women (*p* = 0.69, *p* = 0.58, and *p* = 0.56, resp.). No relationship was found for serum vitamin B12 and folic acid levels regarding gender among all patients and treatment subgroups.

## 4. Discussion

In our study, we found that there were no differences in Hcy values between PD patients treated with entacapone and levodopa and patients treated with levodopa without entacapone. Our results indicated that COMT inhibitors may not have an effect on preventing the potential development of high levels of Hcy due to levodopa.

Hyperhomocysteinemia is a known risk factor for atherosclerotic vascular diseases and dementia [1, 2] and may develop from genetic or acquired causes [3]. Genetic mutations in the C677T allele encoding the MTHFR enzyme are causes of hyperhomocysteinemia, a condition frequently encountered in the general population [3]. Acquired causes include severe metabolic disorders, vitamin B12 and folate deficiency, and the use of vitamins and certain medications [3].

In 1995, Allain et al. were the first to determine that Hcy levels were higher in PD patients than those in healthy subjects [25]. Following this, Müller et al. showed that the Hcy levels of patients treated with long-term levodopa were higher than those in patients who had never used levodopa [26]. In PD patients, plasma Hcy levels may increase because of chronic levodopa therapy. The catabolism of levodopa with the COMT enzyme results in SAH, which rapidly hydrolyses and eventually forms Hcy [3].

Some studies have shown that the combination of levodopa/decarboxylase inhibitors with the COMT inhibitor entacapone lowers the levels of Hcy in rats [9]. While experimental studies have shown that COMT inhibitors may lower Hcy levels, the results of* in vivo* prospective studies with this objective have varied.

Valkovič et al. studied three groups of PD patients treated with the following: (1) levodopa; (2) both levodopa and entacapone; and (3) dopamine agonists. The mean plasma Hcy levels were higher in the levodopa group; in addition, the levodopa group was more prone to B12 hypovitaminosis compared with the other groups [10].

Lamberti et al. studied plasma Hcy levels in treatment groups of levodopa and levodopa-entacapone combination and found that the Hcy levels of both groups were higher than those in the control group, but the folate level was lower in the levodopa group. By statistical analysis, they also found that the lower Hcy levels of the levodopa-entacapone combination group compared with the levodopa group were related to the use of entacapone rather than the effect of folate [11].

In a study performed with four groups of patients (levodopa, levodopa-entacapone combination, dopamine agonist, and control), Zoccolella et al. determined that the levodopa increased plasma Hcy, while the COMT inhibitors such as entacapone effectively reduced this increase [12].

Nevrly et al. compared Hcy levels in patients who used long-term levodopa with Hcy levels in patients who never used levodopa and started the levodopa-entacapone combined treatment. By week 8, there was no increase in the Hcy levels of the patients undergoing combined therapy. Based on this result, they concluded that combination therapy might protect against increases in Hcy in the early stages of PD [27].

Müller and Muhlack showed that while acute levodopa treatment increases plasma Hcy, entacapone prevents this increase. This study highlighted the fact that the effect of entacapone on the Hcy level was acute and that this should be taken into consideration in Hcy measurements [28].

However, while some studies suggest that high levels of Hcy due to levodopa therapy may be prevented with COMT inhibitors, other studies do not support such findings.

O'Suilleabhain et al. concluded that entacapone had no effect on plasma Hcy [13]. Following this study, in a multicentre, open-ended study of 169 patients, patients receiving levodopa therapy were switched to levodopa-entacapone therapy for at least 4 weeks. The results revealed that there was no difference in plasma Hcy levels before and after entacapone use and that entacapone therapy was ineffective in reducing plasma Hcy [14].

In a 6-week, randomized, double-blind, placebo-controlled study, Postuma et al. investigated the effect of 1 mg of folate/500 mg of vitamin B12 and entacapone on serum Hcy in 35 PD patients who were receiving levodopa treatment; entacapone was found to be ineffective compared with placebo and folate/vitamin B12 was found to be effective in reversing the levodopa-related hyperhomocysteinemia [29].

Zesiewicz et al. examined the effect of entacapone use on the elevation of plasma Hcy levels due to levodopa use in PD in a meta-analysis consisting of studies from Europe and the USA. The studies by Lamberti, Zoccolella, and Valkovič showed entacapone use decreased plasma Hcy levels as a positive outcome, but the studies by Ostrem and O'Suilleabhain did not. Zesiewicz et al. underlined patients of three studies showed a decreasing effect of entacapone with low vitamin levels. On the other hand, patients in Ostrem and O'Suilleabhain's study group had normal vitamin levels. Zesiewicz et al. concluded normal or higher vitamin levels may help to metabolize Hcy more and the positive outcome of the European studies could be a result of vitamin levels instead of COMT inhibition [15].

In our study, while there was no difference in plasma Hcy levels between levodopa and levodopa-entacapone combination, Hcy levels were not statistically different in patients who received dopamine agonist alone compared with other groups. However, apart from the group III patients receiving dopamine agonist alone, median Hcy levels in groups I and II were slightly above normal values. These results suggested that levodopa therapy might increase Hcy levels but not significantly; however, concomitant use of entacapone with levodopa might not reduce Hcy levels. In our study, vitamin B12 and folic acid values were normal in all the three groups and there was no difference between groups regarding vitamin B12 and folic acid values. It is possible that levodopa did not cause a significant increase in Hcy levels in our patients because their vitamin levels were normal. As suggested in other studies, the main cause of hyperhomocysteinemia may be low vitamin B12 and/or folic acid levels rather than levodopa treatment [15].

In this study, we found that the duration of PD was shorter in patients treated with dopamine agonist than patients treated with levodopa and entacapone. This result may be explained since dopamine agonists are the first choice of treatment in the earlier period of PD. In addition, when the disease duration advances, progression may occur; therefore, levodopa and COMT inhibitors may take place in the treatment regimen. Data showed that the UPDRS II and III scores were lower in group III than in groups I and II but without statistical significance.

We also found that the onset age was younger in the group treated with levodopa and entacapone compared with patients treated with levodopa and with the group treated with the dopamine agonist alone. This may be because when the age at the disease onset was earlier, more disease progression and motor fluctuations may appear in the advanced disease; therefore, levodopa and COMT inhibitors usually were administered in this group.

Some studies have suggested a correlation between hyperhomocysteinemia and male gender, but this correlation has not been demonstrated in other studies [5, 30–32]. We compared plasma Hcy levels between men and women and found that plasma Hcy levels were higher in men, but we could not find any statistical difference between treatment subgroups of male patients and similarly in female subgroups. In addition, there was no difference according to gender distribution in the subgroups in our study. Therefore, gender may not have been an important factor on plasma Hcy levels in the results of this study.

## 5. Conclusions

The results of our study indicated that, in PD patients, levodopa therapy may cause slight increases in plasma Hcy levels, though not a significant increase, when compared with dopamine agonists. Furthermore, the results did not support the finding that COMT inhibitors have a preventive effect on hyperhomocysteinemia, which may be associated with levodopa therapy.

## Figures and Tables

**Table 1:** Demographic data, clinical characteristics, and serum vitamin B12, folic acid, and homocysteine levels of patients^*∗*^.

	(*n* = 100)
Age (years)	70 (40–89)
Male/female	54 (54%)/46 (46%)
Duration of PD (years)	5 (1–24)
Age of onset (years)	65 (32–86)
UPDRS II score	10 (2–36)
UPDRS III score	15 (6–43)
mHY stage	2 (1–5)
SMMT score	24 (10–30)
Hcy (*µ*mol/L)	14.45 (5.18–54.3)
Vitamin B12 (pg/mL)	263.5 (58–990)
Folic acid (ng/mL)	9.29 ± 3.44

^*∗*^Data reported as median (minimum–maximum) except folic acid values. Folic acid values reported as mean ± SD.

PD: Parkinson's disease; UPDRS: Unified Parkinson's Disease Rating Scale; mHY: modified Hoehn and Yahr staging scale; SMMT: Standardized Mini-Mental Test; Hcy: homocysteine.

**Table 2:** Demographic data, clinical characteristics, and serum vitamin B12, folic acid, and homocysteine levels of patients with PD according to treatment groups^*∗*^.

	Group I (*n* = 58)	Group II (*n* = 30)	Group III (*n* = 12)	*p*
Age (years)	70 (40–89)	65.5 (42–84)	69.5 (53–77)	0.04^†^
Male/female	32 (59.3%)/26 (56.5%)	16 (29.6%)/14 (30.4%)	6 (11.1%)/6 (13%)	0.94
Duration of the PD (years)	5 (1–20)	9.5 (1–24)	3 (1–12)	0.01^‡^
Age of onset (years)	65 (35–86)	57 (32–78)	67 (52–72)	0.002^§^
UPDRS II score	11 (3–36)	12 (3–26)	8 (2–16)	0.07
UPDRS III score	15 (6–43)	17 (6–35)	14 (6–30)	0.27
mHY stage	2 (1–5)	2 (1–5)	1.5 (1–3)	0.23
SMMT score	24 (10–30)	25 (15–29)	26 (17–28)	0.46
Hcy (*µ*mol/L)	15.1 (6.3–50)	15.2 (5.18–36)	12.6 (7.92–54.3)	0.30
Vitamin B12 (pg/mL)	268.1 (100–662)	262.5 (147.9–990)	295 (58–768)	0.80
Folic acid (ng/mL)	9.38 ± 2.87	8.67 ± 3.67	10.73 ± 5.02	0.26
LD dose (mg/day)	300 (100–1000)	400 (150–1000)	(—)	0.30
Duration of LD medication (years)	2.5 (1–20)	5.5 (1–20)	(—)	0.27
COMTI dose (mg/day)	(—)	800 (400–1400)	(—)	(—)
Duration of COMTI medication (years)	(—)	2 (1–10)	(—)	(—)

^*∗*^Data reported as median (minimum–maximum) except folic acid values. Folic acid values reported as mean ± SD.

^†^Statistical difference was significant between group I and group II (*p *< 0.05).

^‡^Statistical difference was significant between group II and group III (*p *< 0.05).

^§^Statistical difference was significant between group II and both of the other groups (*p *< 0.05).

PD: Parkinson's disease; UPDRS: Unified Parkinson's Disease Rating Scale; mHY: modified Hoehn and Yahr staging scale; SMMT: Standardized Mini-Mental Test; Hcy: homocysteine; LD: levodopa; COMTI: catechol-O-methyl transferase inhibitors.
